# Service Availability and Readiness of Primary Care Health Facilities Offering Hypertension Diagnosis Services in Wakiso District, Uganda, 2019

**DOI:** 10.5888/pcd20.220236

**Published:** 2023-03-30

**Authors:** Jackline Nanono, Dinesh Neupane, Tonny Ssekamatte, Emmanuel Ahumuza, Francis Xavier Kasujja, Elizeus Rutebemberwa

**Affiliations:** 1 Makerere University, School of Public Health, Kampala, Uganda; 2 Department of International Health, Johns Hopkins Bloomberg School of Public Health, Baltimore, Maryland; 3 Department of Epidemiology and Biostatistics, Makerere University College of Health Sciences, Kampala, Uganda; 4 The Chronic Diseases and Cancer Theme, Medical Research Council/Uganda Virus Research Institute and London School of Hygiene & Tropical Medicine Research Unit, Entebbe, Uganda

## Abstract

**Introduction:**

Hypertension is a growing burden in Uganda and other low- and middle-income countries. Appropriate diagnosis services are needed at primary care health facilities to identify, initiate treatment for, and manage hypertension. This study assessed service availability and readiness as well as facilitators and barriers in primary health care facilities for hypertension diagnosis services in Wakiso District, Uganda.

**Methods:**

In July and August 2019, we conducted structured interviews at 77 randomly selected primary care health facilities in Wakiso District. We used an interviewer-administered health facility checklist modified from the World Health Organization’s service availability and readiness assessment tool. We also conducted 13 key informant interviews with health workers and district-level managers. Readiness was measured by availability of functional diagnostic equipment, related supplies and tools, and health provider attributes. Service availability was measured by assessing hypertension diagnosis services.

**Results:**

Most (86%; 66 of 77) health facilities offered hypertension diagnosis services and 84% (65 of 77) had digital blood pressure measuring devices; only 69% (53 of 77) had functional blood pressure measuring devices. Lower-level facilities lacked appropriate blood pressure cuffs for use across age groups: 92% (71 of 77) lacked pediatric cuffs and 52% (40 of 77) lacked alternative adult cuﬀs. Facilitators for diagnosing hypertension included partners that built health facility staff capacity and funds for purchasing hypertension diagnostic supplies; common barriers were nonfunctional equipment, delays in receiving training, and inadequate staffing.

**Conclusion:**

The results highlight the need for an adequate supply of devices, routine replacements or repairs, and frequent refresher training for health workers.

**Summary**

**What is already known on this topic?**

Hypertension is the most common cardiovascular disease in the world. If left uncontrolled, it can cause stroke, myocardial infarction, cardiac failure, renal failure, and hypertensive retinopathy. Diagnosis is the first step in managing and treating hypertension. The condition is highly undiagnosed in sub-Saharan Africa because screening programs are limited.

**What is added by this report?**

In Uganda, most primary health care facilities offer diagnostic services, but few have functioning diagnostic equipment, and refresher training is rarely available for health workers.

**What are the implications for public health practice?**

If diagnostic services are not available on a timely basis, the burden of hypertension will continue to increase in Uganda.

## Introduction

Hypertension is the most common cardiovascular disease and a leading risk factor in illness and death throughout the world. Globally, in 2021, 1.28 billion people aged 30 to 79 years had hypertension, two-thirds of whom lived in low- and middle-income countries (1). Diagnosis is the first step in hypertension care. Recommended items for the thorough measurement of hypertension are sphygmomanometers (blood pressure monitors) with appropriately sized cuffs, stadiometers (to measure height), and weighing machines; assessment of hypertension risks or appropriate referrals by health care providers is also recommended (2,3).

Despite knowledge about the growing hypertension epidemic, few people receive adequate diagnostic services in primary care facilities in low- and middle-income countries (4). In sub-Saharan Africa, screening and early diagnosis programs are limited; thus, hypertension is largely undiagnosed. A systematic review and meta-analysis to assess the prevalence of hypertension in sub-Saharan Africa examined data from 33 surveys and found that an average of 27% of people were aware of their hypertensive status before the surveys (5). People with hypertension need proper diagnosis, frequent monitoring, and lifelong treatment, which is a challenge for Uganda, a low-income, resource-constrained country (6,7). Failure to diagnose hypertension in a timely way exposes people to an increased risk of cardiovascular disease, which further burdens already insufficient health care resources (7).

In Uganda, health services are delivered within a framework of decentralization; 4 health facility levels offer specified services to a designated population and catchment area. The diagnosis of hypertension is made at all health facility levels, including Health Centre II (HCII), Health Centre III (HCIII), and Health Centre IV (HCIV), despite the Ministry of Health mandate that HCIIs are not supposed to manage patients with hypertension (8).

Uganda faces a budding epidemic of hypertension. In 2015, the prevalence of hypertension was 26.4%, and the condition remains undiagnosed and undetected in many people (9). A study conducted in 1 district in central Uganda reported that 6.3% of people in Uganda self-reported having hypertension (10). Wakiso District has the largest population in central Uganda, with 2.9 million people. The estimated prevalence of hypertension is 34.3% in this region, the highest of any region in Uganda (11).

The available information on hypertension in Uganda in the scientific literature mostly describes the proportion of people at risk for hypertension and those who have hypertension (9–11). Few published studies focus on the status of hypertension diagnostic services available in Uganda and the readiness of health facilities to offer hypertension care (12). The objective of our study was to describe this availability and readiness and the facilitators and barriers for hypertension diagnosis in health facilities in the Wakiso District. Findings from our study will provide policy makers, health care providers, and nongovernment organizations (NGOs) (eg, Medical Research Council, MildMay, Rotary International) with information about the existing services and readiness of health facilities to provide hypertension diagnostic services and identify bottlenecks and solutions to improve service delivery.

## Methods

### Study area and study population

Wakiso District, which partly encircles Kampala, the capital district of Uganda, has 70 public health facilities and 38 private, nonprofit health facilities. The study population was selected from both types of health facilities, and included HCIIs, HCIIIs, HCIVs, and hospitals. An HC-II is found at the parish level and offers preventive, promotive, outpatient curative health services, outreach care, and emergency services to a population of approximately 5,000. An HCIII is found at the subcounty level and offers all services offered at HCIIs, plus maternity, inpatient, and laboratory services to a population of approximately 20,000. An HCIV is found at the county level and offers all services offered at HCIIIs, plus emergency surgery and blood transfusion to a population of approximately 100,000. A hospital is also found at the district level and offers all services offered at HCIVs, plus service training, consultation, and research to a population of approximately 500,000.

### Study design

This cross-sectional study used both quantitative and qualitative approaches and was conducted in July and August 2019. Quantitative data were collected on service availability and readiness of health facilities. Qualitative data were collected on the facilitators and barriers faced by health workers while providing hypertension diagnostic services.

### Operational definitions

Service availability was measured by assessing the provision of blood pressure measurements, scheduling of regular checkups by health workers, referrals, access to transportation to access the referral health facility, and the management of patients with suspected or newly diagnosed cases of hypertension.

Service readiness was measured by assessing the presence and functional status of blood pressure devices, including the availability of various cuff sizes for use across age ranges. It also included the availability of a private room or confidential area for counseling and taking measurements for hypertension diagnosis; a book or other means for routine reporting; the presence of standard guidelines; access to hypertension training and continuing medical education sessions; the availability of a health worker at the health facility 24 hours per day or on-call to screen, diagnose, or make referrals; and a point person for ordering hypertension diagnostic supplies and equipment.

### Sample size and assumptions

For the quantitative study, we assumed a design effect of 1.2 and a 7% nonresponse rate. We calculated the sample size as 79 health facilities (13); we removed 2 facilities from the analysis because we found that 1 HCII was no longer functioning and 1 HCIII no longer existed. Thus, we analyzed data from 77 health facilities, including 34 HCII-level facilities, 31 HCIII-level facilities, 7 HCIV-level facilities, and 5 hospitals. We also interviewed 13 health workers and district health managers for qualitative data. For these interviews, we spoke with 4 staff members from HCII-level facilities, 4 staff members from HCIII-level facilities, 1 staff member from an HCIV-level facility, 1 staff member from 1 of the 5 hospitals, and 3 staff members from district health offices.

### Sampling

From a sample of 108 health facilities, we randomly selected facilities by stratification from each health facility level. We selected every third HCII- and HCIII-level facility and all HCIV-level facilities and hospitals. With guidance from health facility managers, we purposely chose 1 health worker from each health facility. For the key informant interviews, personnel were selected from health facility levels and district health offices.

### Data collection tools

We developed a checklist based on the service availability and readiness assessment tool developed by the World Health Organization (WHO) to collect information on service availability and readiness of health facilities. The checklist was modified to include only information relating to hypertension screening and diagnosis, but it maintained consistency with other variables in the WHO tool.

This checklist was administered to the managers at all sampled health facilities (13). The semistructured interview guide was also administered to 1 health worker from each sampled health facility. For the key informant interviews, we developed an interview guide to focus on policy issues at the health facility and district levels.

### Data collection

All data were collected by interviewers who were trained for 4 days and had at least 4 years of experience in data collection. All data collection tools were administered face-to-face by the interviewers, with sessions lasting from 30 to 50 minutes. The information from the interview checklist and the semistructured tools was collected on Android mobile telephones by using the KoboCollect application version 2.019.22 (Kobo Inc), while the key informant interviews were audio recorded. All interviews were conducted in confidential spaces.

### Data analysis

Data were retrieved from the KoboCollect server, exported into an Excel (Microsoft Corporation) spreadsheet, and analyzed with Stata version 14 (StataCorp, LLC). Weights were scored as 1, for what was available at the health facility, and 0, for what was absent (13–15). As adopted from the service availability and readiness assessment reference manual, the responses in each domain were aggregated into an index of composite scores for categories of readiness. We conducted χ^2^ tests and Fisher exact tests to explore the association between health facility levels and variables relating to the availability and functional status of diagnostic equipment and tools. We considered χ^2^ *P* values of less than .05 significant. For the qualitative component, the key informant interview audio files were transcribed verbatim and checked for consistency before field notes were compiled. Meaningful units were generated from the text and condensed into codes. The codes were generated and later merged into themes. The themes were organized, grouped, and checked for consistency before manual thematic analysis was performed.

### Ethical considerations

Written informed consent was obtained from all participants and documented on approved forms. Study approval was obtained from the Uganda National Council of Science and Technology through the institutional review board of the Makerere University School of Public Health Higher Degrees Research and Education Committee. Permission to conduct the research was obtained from the district health office of Wakiso District and the medical superintendent of Entebbe Hospital.

## Results

Of the 77 health facilities examined, 90% (n = 69) measured the blood pressure of patients, 86% (n = 66) offered comprehensive hypertension diagnosis services, and 79% (n = 61) scheduled regular blood pressure checkups. We found significant differences in managing suspected or newly diagnosed cases of hypertension (*P* = .001) and access to means of transportation for referral across the different health facility levels (*P* = .001) (Table 1).

**Table 1:** Services Available for Hypertension Diagnosis Across Different Levels of Primary Care Health Facilities, Wakiso District, Uganda, 2019

Variable	Health facility level, no. (%) (N = 77)				*P* value^e^
	Health Centre II^a^ (n = 31)	Health Centre III^b^ (n = 34)	Health Centre IV^c^ (n = 7)	Hospital^d^ (n = 5)	
Offers diagnosis of hypertension	20 (64.5)	34 (100)	7 (100)	5 (100)	.001
Takes blood pressure measurements	23 (74.2)	34 (100)	7 (100)	5 (100)	.005
Takes weight measurements	26 (83.9)	34 (100)	7 (100)	5 (100)	.07
Offers motivational counseling	26 (83.9)	33 (97.1)	7 (100)	5 (100)	.25
Manages suspected or newly diagnosed cases of hypertension	11 (35.5)	32 (94.1)	7 (100)	5 (100)	.001
Schedules regular checkups	18 (58.1)	31 (91.2)	7 (100)	5 (100)	.003
Has a referral system	27 (87.1)	26 (76.5)	1 (14.3)	1 (20)	.001
Has access to transportation for referral	3 (9.7)	12 (35.3)	3 (42.9)	5 (100)	.001

a Found at the parish level and offers preventive, promotive, outpatient curative health services, outreach care, and emergency services to a population of approximately 5,000.

b Found at the subcounty level and offers all services offered at HCIIs, plus maternity, inpatient, and laboratory services to a population of approximately 20,000.

c Found at the county level and offers all services offered at HCIIIs, plus emergency surgery and blood transfusion to a population of approximately 100,000.

d Found at district level and offers all services offered at HCIVs, plus service training, consultation, and research to a population of approximately 500,000.

e Determined by χ^2^ test; *P* value <.05 considered significant.

### Hypertension diagnostic equipment and related supplies

Most lower-level primary care health facilities lacked a range of blood pressure cuffs for use across age groups; 92% (71 of 77) lacked pediatric blood pressure cuffs, and 52% (40 of 77) lacked alternative adult blood pressure cuﬀs (Table 2).

**Table 2:** Hypertension Diagnostic Equipment and Related Supplies Across Different Levels of Primary Care Health Facilities, Wakiso District, Uganda, 2019

Variable	Health facility level, no. (%) (N = 77)				*P* value^e^
	Health Centre II^a^ (n = 31)	Health Centre III^b^ (n = 34)	Health Centre IV^c^ (n = 7)	Hospital^d^ (n = 5)	
Available digital sphygmomanometer	23 (74.2)	30 (88.2)	7 (100.0)	5 (100.0)	.26
Available mercury sphygmomanometer	17 (54.8)	25 (73.5)	4 (57.1)	4 (80.0)	.36
Available aneroid sphygmomanometer	15 (48.4)	20 (58.8)	3 (42.9)	5 (100)	.16
Functional digital sphygmomanometer	18 (58.1)	25 (73.5)	5 (71.4)	5 (100.0)	.25
Functional mercury sphygmomanometer	6 (19.4)	19 (55.9)	2 (28.6)	2 (40.0)	.02
Functional aneroid sphygmomanometer	6 (19.4)	19 (55.9)	2 (28.6)	4 (80.0)	.003
Available adult standard blood pressure cuffs (25 cm ×12 cm)	27 (87.1)	32 (94.1)	7 (100.0)	5 (100.0)	.45
Available alternative blood pressure cuffs (36 cm × 12 cm)	9 (29.0)	17 (50.0)	6 (85.7)	5 (100.0)	.002
Available pediatric blood pressure cuffs	2 (6.5)	2 (5.9)	0	2 (40.0)	.12
Available body mass index charts	11 (35.5)	15 (44.1)	6 (85.7)	3 (60.0)	.14
Available guidelines on hypertension diagnosis	29 (93.5)	30 (88.2)	7 (100.0)	5 (100.0)	.89
Job aids available on hypertension diagnosis	16 (51.6)	24 (70.6)	6 (85.7)	5 (100.0)	.04
Available height-measuring tape, board, or stadiometer	14 (45.2)	28 (82.4)	7 (100.0)	5 (100.0)	.001
Available automated weighing machine	5 (16.1)	9 (26.5)	3 (42.9)	5 (100.0)	.29
Available manual weighing scale	23 (74.2)	33 (97.1)	5 (71.4)	2 (40.0)	.02
Functional automated weighing machine	4 (12.9)	7 (20.6)	3 (42.9)	2 (40.0)	.17
Functional manual weighing scale	22 (71.0)	33 (97.1)	5 (71.4)	5 (100.0)	.04
Available source document used for monthly reporting	20 (64.5)	30 (88.2)	7 (100.0)	5 (100.0)	.04

a Found at the parish level and offers preventive, promotive, outpatient curative health services, outreach care, and emergency services to a population of approximately 5,000.

b Found at the subcounty level and offers all services offered at HCIIs, plus maternity, inpatient, and laboratory services to a population of approximately 20,000.

c Found at the county level and offers all services offered at HCIIIs, plus emergency surgery and blood transfusion to a population of approximately 100,000.

d Found at district level and offers all services offered at HCIVs, plus service training, consultation, and research to a population of approximately 500,000.

e Determined by χ^2^ test; *P* value <.05 considered significant.

### Health workers’ related practices on hypertension diagnosis

Refresher training on hypertension diagnosis had not been given to 83% (64 of 77) of health facilities. One HCII-level facility did not have a confidential area for diagnosis. We found significant differences in diagnosis practices by health facility level: provision of health care provider refresher training on hypertension diagnosis in the last 6 months (*P* = .002); 24-hour availability of a health care provider at the health facility (*P* = .001) to screen, diagnose, or make a referral; and a point person for ordering hypertension diagnostic supplies (*P* = .01) (Table 3).

**Table 3:** Hypertension Diagnosis–Related Practices Among Health Workers Across Different Levels of Primary Care Health Facilities, Wakiso District, Uganda, 2019

Variable	Health facility level, no. (%) (N = 77)				*P* value^e^
	Health Centre II^a^ (n = 31)	Health Centre III^b^ (n = 34)	Health Centre IV^c^ (n = 7)	Hospital^d^ (n = 5)	
At least 1 health care provider received refresher training in the diagnosis of hypertension in the last 6 months	2 (6.5)	5 (14.7)	5 (71.4)	1 (20.0)	.002
Health care providers who are attached and posted to the health facility to diagnose hypertension	21 (67.7)	34 (100.0)	7 (100.0)	5 (100.0)	.001
Trained health care provider available at the facility on call, 24 hours per day, including weekends, and on public holidays to screen, diagnose, or make referrals	5 (16.1)	27 (79.4)	7 (100.0)	5 (100.0)	.001
Private room or confidential area for hypertension diagnosis and counseling with audio and visual privacy	30 (96.8)	34 (100.0)	7 (100.0)	5 (100.0)	.56
Point person for ordering hypertension diagnostic supplies and equipment	15 (48.4)	27 (79.4)	7 (100.0)	5 (100.0)	.01

a Found at the parish level and offers preventive, promotive, outpatient curative health services, outreach care, and emergency services to a population of approximately 5,000.

b Found at the subcounty level and offers all services offered at HCIIs, plus maternity, inpatient, and laboratory services to a population of approximately 20,000.

c Found at the county level and offers all services offered at HCIIIs, plus emergency surgery and blood transfusion to a population of approximately 100,000.

d Found at district level and offers all services offered at HCIVs, plus service training, consultation, and research to a population of approximately 500,000.

e Determined by χ^2^ test; *P* value <.05 considered significant.

### Facilitators and barriers to providing hypertension diagnostic services

Multiple facilitators and barriers of hypertension diagnostic services were cited during key informant interviews (Table 4). Of the 10 key informant interviews conducted with staff members at the various levels of health facilities, 7 interviewees mentioned that NGOs were actively involved in providing equipment. “Like the supply of equipment is supposed to be done by the ministry, but very often we squeeze from the Primary Health Care fund, [and] we buy a few” [key informant no. 9, district health team member].

**Table 4:** Facilitators and Barriers in Providing Hypertension Diagnosis Services Across Different Levels of Health Facilities, Wakiso District, Uganda, 2019

Health facility level	Facilitators to hypertension diagnosis services	Barriers to hypertension diagnosis services
Hospital^a^	• Medical training • Equipment availability • Clinical experience • Continuous medical education	• Lack consistent funding for village follow-ups • Patients miss scheduled appointments • Difficulties in measuring height of women with plaited hair • Lack measures to investigate other comorbidities • Resistance from patients to take measurements
Health Centre IV^b^	• Equipment availability • Implementing partners • History-taking and assessments of hypertension diseases in the patient’s family • Continuous medical education	• Lack supplies or batteries for digital equipment • Lack measures of investigation of other comorbidities • Faulty equipment • Lack consistent funding for village follow-ups • Contradicting information on hypertension from different guidelines
Health Centre III^c^	• Equipment availability • Clinical experience • History-taking and assessments of hypertension diseases in the patient’s family • Medical training • Guidelines • Triage of patients • Responsive patients • Continuous medical education • On-the-job training • Hypertension treatment cards • Presence of other health workers who provide mentorship	• Lack consistent funding for village follow-ups • Faulty equipment • Resistance from patients to take measurements • Patients miss scheduled appointments • Lack supplies or batteries for digital equipment • Blood pressure cuffs do not fit patients’ arms • Blood pressure apparatus rarely calibrated • Time-consuming to take several measurements • Lack measures to investigate other comorbidities • Difficult to use the manual blood pressure machine • Lack of hypertension cards • Uncertainty about referrals made for patients to reach higher-level health facilities • Lack of equipment
Health Centre II^d^	• Equipment availability • History-taking and assessments of hypertension diseases in the patient’s family • Clinical experience • Implementing partners • Triage of patients • Responsive patients • Medical training	• Uncertainty about referrals made for patients to reach higher-level health facilities • Faulty equipment • Lack supplies or batteries for digital equipment • Lack consistent funding for village follow-ups • Patients miss scheduled appointments • Blood pressure cuffs do not fit patient’s arm • Difficult to use the manual blood pressure machine

a Found at district level and offers all services offered at HCIVs, plus service training, consultation, and research to a population of approximately 500,000.

b Found at the county level and offers all services offered at HCIIIs, plus emergency surgery and blood transfusion to a population of approximately 100,000.

c Found at the subcounty level and offers all services offered at HCIIs, plus maternity, inpatient, and laboratory services to a population of approximately 20,000.

d Found at the parish level and offers preventive, promotive, outpatient curative health services, outreach care, and emergency services to a population of approximately 5,000.

One informant mentioned that NGOs were involved in building capacity for health workers. Primary health care funds were another facilitator mentioned by 9 informants; these funds were used to purchase hypertension diagnostic supplies and some equipment. Twelve informants mentioned the availability of referral services, noting that patients at lower-level health facilities were referred to higher-level health facilities when required. “[We] take the blood pressure . . . and then after that, you advise accordingly because this is a HCII whereby we are not supposed to prescribe for such; we monitor like some days . . . but if [blood pressure is] very high we refer them” [Key informant no. 4, enrolled nurse, HCII].

The key barrier reported by 9 informants was not having functional blood pressure equipment. Ten informants noted that equipment was defective; they said that the machines gave them inaccurate measurements and other equipment was not working at all. Three informants reported that there were few blood pressure devices in their health facilities. Three informants noted the struggle to acquire batteries and the lack of batteries for the digital hypertension diagnostic equipment. Three informants reported a lack of information on where repairs and calibrations could be done on nonfunctional and faulty equipment: “In fact, we don’t know where equipment could be repaired, I would personally take them because I am the in-charge, but I don’t know where” [Key informant no. 6, midwife, HCII].

Two HCII informants noted that their facility received no hypertension diagnostic equipment because of the Ministry of Health mandate that HCIIs are not supposed to manage patients with hypertension. Two health workers reported that insufficient manpower hindered the ability to carry out hypertension screening and other measurements. A concern was raised by 6 informants about the lack of refresher training and not having the latest information about hypertension diagnosis. The lack of continuing medical education, because of limited funds, was noted by 2 informants. Four informants mentioned a lack of knowledge and skills to operate equipment and that newly recruited health workers are transferred without being trained.

They [continuing medical education opportunities] depend on the funding; if we have funds, we will be trained because you can’t conduct a training without funds. So, their frequencies are dictated by the amount of money; also, there’s knowledge gaps especially in lower levels; here we have a sector only with nurses, and sometimes there are knowledge gaps [key informant no. 11, district health office].

## Discussion

The findings from our study highlight that many health facilities at different levels of the primary care health system in Wakiso District provide some form of hypertension diagnostic services. However, the availability and readiness of diagnostic services vary significantly. Several health facilities had all necessary supplies and tools for hypertension diagnosis, while other facilities had no equipment or had broken equipment. The key informants noted the infrequency of continuing medical education or refresher training opportunities. Implementing partners and the government’s primary health care funds were key in mitigating some of the barriers faced in providing hypertension diagnosis services.

Our study showed that health facilities delivered hypertension diagnostic services to patients. However, the combination of several services for hypertension diagnosis across different levels of the health system was not comprehensive. This finding is consistent with the findings of similar studies carried out in Tanzania and other areas of Uganda (14,16).

Many primary care health facilities had hypertension diagnostic equipment, but some of the equipment was not functional. This finding concurs with those of a similar study done in Tanzania: although more than half of the health facilities in that study had hypertension diagnostic equipment, some of it was faulty (14). As a result of the Ministry of Health mandate that HCIIs are not supposed to manage patients with hypertension, some lower-level health facilities lacked diagnostic equipment, and others did not receive adequate supplies and personnel. Therefore, even though hypertension diagnosis services could be offered at HCII-level facilities and other level health facilities, the lack of vital functioning equipment prevented availability and readiness of service provision.

One-third of HCII-level facilities had the capacity to manage patients with suspected hypertension. This finding could be explained by the Ministry of Health’s mandate that HCIIs are not supposed to manage patients with hypertension but can only refer patients (8,17). This finding is similar to the finding of a study in Tanzania that highlighted the weak management, training, and reporting systems and the complex ordering process for basic medicines and equipment, which affected the health facilities’ preparedness to manage patients with hypertension (14). Health facilities that do not receive adequate supplies and personnel are hindered from providing hypertension diagnosis services to patients with probable hypertension.

Key informant interviews pointed to inconsistencies in the presence and functional status of equipment for diagnosing hypertension, especially blood pressure machines, an essential requirement for diagnosing hypertension. These findings are supported by those of another study, conducted in Uganda at high-level health facilities, on the availability of equipment needed to manage noncommunicable diseases (12). Because of broken or unavailable blood pressure machines, many health workers at primary care health facilities cannot offer hypertension diagnosis services to patients.

Most health facilities in our study lacked cuffs appropriate for measuring blood pressure among patients ranging from children to mid-sized adults. The lack of different blood pressure cuff sizes could explain why some health workers in our study reported challenges in taking blood pressure measurements. Another study conducted in Uganda reported a 70% deficiency of standard cuffs and a scarcity of pediatric cuffs (16). Standard adult cuffs are too small for about one-third of patients; an inappropriately small size causes variability in blood pressure readings and leads to underdiagnosis or overdiagnosis of hypertension (18–20).

Only approximately one-third of the health facilities in our study had access to transportation for referred patients. This proportion is similar to that found in another study conducted in Uganda at higher-level health facilities (regional referral hospitals, general hospitals, and HCIVs in the public sector): more than half of patients referred to another facility for diagnosis or management lacked access to an ambulance or other means of transport (12,21). These findings imply that health workers face difficulties in finding means of transportation to referral centers and other high-level health facilities, and that lack of transportation can be a barrier to managing hypertension.

Our study also highlighted that most health facilities offered counseling on behavioral modifications for patients with hypertension. This finding contrasted with the findings of studies in Uganda and Zimbabwe, where some health care providers reported not providing hypertension and diabetes education plus counseling because they lacked information, proper training, and the time required for such education and counseling (15,16). Counseling can act as a mode of behavior change and encouragement for reducing health risks among clients and prospective patients (20,22). Several studies from Asia have demonstrated that nonphysician health workers can be trained to diagnose hypertension (23,24), and sharing or shifting tasks among health workers can help to solve the problem of lack of staff members capable of and available for diagnosing hypertension. This finding implies that health workers and nonphysicians, when equipped with updated information and functional equipment, can diagnose hypertension, assess hypertension risks, and motivate behavior change among patients.

### Strengths and limitations

Our study is the first to document service availability and readiness of primary care health facilities to offer hypertension diagnosis services in Wakiso District, Uganda. We used previously validated WHO tools and collected data from different types of health facilities. We modified the tool to include only variables relating to hypertension screening and diagnosis. Thus, the validity of the WHO tool was maintained. We used mixed methods to triangulate findings. Although the study was confined to Wakiso District, the results could be generalized to other districts in Uganda with similar circumstances and other countries in sub-Saharan Africa with similar health systems.

Limitations of the study included focusing only on the availability and readiness of hypertension diagnostic services and not including drug availability for managing or treating patients with hypertension. The participants who were chosen from purposively selected health facilities were few.

### Conclusion

Most primary care health facilities in Wakiso District offered hypertension diagnosis services. Yet we found significant differences in the availability and functional status of the hypertension diagnostic equipment. Health facilities across all levels should be supported to offer quality hypertension diagnostic services by the Wakiso District Health Office and the Ministry of Health. By providing essential equipment, and supporting routine repairs and maintenance of damaged equipment, service provision will be increased and enhanced across all health facility levels. Further studies are needed to explore the health service delivery of hypertension diagnosis services across urban, suburban, and rural health facilities, and across private for-profit, private nonprofit, and public health facilities to clarify where hypertensive patients seek the most care.

## References

[1] World Health Organization. Hypertension fact sheet. August 25, 2021. Accessed June 15, 2022. https://www.who.int/news-room/fact-sheets/detail/hypertension

[2] World Health Organization. World Health Organization global status report on noncommunicable diseases, 2014. Accessed July 19, 2022. https://apps.who.int/iris/bitstream/handle/10665/148114/9789241564854_eng.pdf

[3] World Health Organization. Package of essential noncommunicable (PEN) disease interventions for primary health care. 2010. Accessed June 13, 2022. https://www.who.int/publications/i/item/9789240009226

[4] World Health Organization. HEARTs: technical package for cardiovascular disease management in primary health care: systems for monitoring 2018. Accessed June 17, 2021. https://apps.who.int/iris/bitstream/handle/10665/260423/WHO-NMH-NVI-18.5-eng.pdf?sequence=1&isAllowed=y

[5] Ataklte F , Erqou S , Kaptoge S , Taye B , Echouffo-Tcheugui JB , Kengne AP . Burden of undiagnosed hypertension in sub-Saharan Africa: a systematic review and meta-analysis. Hypertension 2015;65(2):291–8. 25385758 10.1161/HYPERTENSIONAHA.114.04394

[6] Ssinabulya I , Nabunnya Y , Kiggundu B , Musoke C , Mungoma M , Kayima J . Hypertension control and care at Mulago Hospital ambulatory clinic, Kampala-Uganda. BMC Res Notes 2016;9(1):487. 10.1186/s13104-016-2293-y 27855717 PMC5114775

[7] Ouyang H . Africa’s top health challenge: cardiovascular disease. The Atlantic. Published October 30, 2014. Accessed July 10, 2022. https://www.theatlantic.com/health/archive/2014/10/africas-top-health-challenge-cardiovascular-disease/381699

[8] The Republic of Uganda Ministry of Health. Uganda Service Provision Assessment Survey 2007. Accessed August 14, 2020. https://dhsprogram.com/pubs/pdf/SPA13/SPA13.pdf

[9] Guwatudde D , Mutungi G , Wesonga R , Kajjura R , Kasule H , Muwonge J , The epidemiology of hypertension in Uganda: findings from the National Non-Communicable Diseases Risk Factor Survey. PLoS One 2015;10(9):e0138991. 10.1371/journal.pone.0138991 26406462 PMC4583385

[10] Siddharthan T , Kalyesubula R , Morgan B , Ermer T , Rabin TL , Kayongo A , The rural Uganda non-communicable disease (RUNCD) study: prevalence and risk factors of self-reported NCDs from a cross sectional survey. BMC Public Health 2021;21(1):2036. 10.1186/s12889-021-12123-7 34743687 PMC8572568

[11] Lunyera J , Kirenga B , Stanifer JW , Kasozi S , van der Molen T , Katagira W , Geographic differences in the prevalence of hypertension in Uganda: results of a national epidemiological study. PLoS One 2018;13(8):e0201001. 10.1371/journal.pone.0201001 30067823 PMC6070243

[12] Rogers HE , Akiteng AR , Mutungi G , Ettinger AS , Schwartz JI . Capacity of Ugandan public sector health facilities to prevent and control non-communicable diseases: an assessment based upon WHO-PEN standards. BMC Health Serv Res 2018;18(1):606. 10.1186/s12913-018-3426-x 30081898 PMC6080524

[13] World Health Organization. Service availability and readiness assessment (SARA): an annual monitoring system for service delivery: reference manual. 2015. Accessed May 20, 2022. https://cdn.who.int/media/docs/default-source/service-availability-and-readinessassessment(sara)/sara_reference_manual_chapter3.pdf?sfvrsn=7a0f3a33_3

[14] Bintabara D , Mpondo BCT . Preparedness of lower-level health facilities and the associated factors for the outpatient primary care of hypertension: evidence from Tanzanian national survey. PLoS One 2018;13(2):e0192942. 10.1371/journal.pone.0192942 29447231 PMC5814020

[15] Mungati M , Manangazira P , Takundwa L , Gombe NT , Rusakaniko S , Tshimanga M . Factors affecting diagnosis and management of hypertension in Mazowe District of Mashonaland Central Province in Zimbabwe: 2012. BMC Cardiovasc Disord 2014;14(1):102. 10.1186/1471-2261-14-102 25135002 PMC4142459

[16] Musinguzi G , Bastiaens H , Wanyenze RK , Mukose A , Van Geertruyden JP , Nuwaha F . Capacity of health facilities to manage hypertension in Mukono and Buikwe districts in Uganda: challenges and recommendations. PLoS One 2015;10(11):e0142312. 10.1371/journal.pone.0142312 26560131 PMC4641641

[17] The Republic of Uganda Ministry of Health. National medicines policy. 2015. Accessed June 1, 2022. https://www.nda.or.ug/wp-content/uploads/2022/02/National-Medicines-Policy-2015-6.pdf

[18] Bao W , Threefoot SA , Srinivasan SR , Berenson GS . Essential hypertension predicted by tracking of elevated blood pressure from childhood to adulthood: the Bogalusa Heart Study. Am J Hypertens 1995;8(7):657–65. 10.1016/0895-7061(95)00116-7 7546488

[19] Rush C , Wauters J , Porter S , Chamie C , Conn B . Improving the screening, prevention, and management of hypertension: an implementation tool for clinic practice teams. Washington State Department of Health; 2013. Accessed June 1, 2022. https://www.healthit.gov/sites/default/files/13_bptoolkit_e13l.pdf

[20] Vos LE , Oren A , Bots ML , Gorissen WH , Grobbee DE , Uiterwaal CS . Does a routinely measured blood pressure in young adolescence accurately predict hypertension and total cardiovascular risk in young adulthood? J Hypertens 2003;21(11):2027–34. 10.1097/00004872-200311000-00011 14597845

[21] Chang H , Hawley NL , Kalyesubula R , Siddharthan T , Checkley W , Knauf F , Challenges to hypertension and diabetes management in rural Uganda: a qualitative study with patients, village health team members, and health care professionals. Int J Equity Health 2019;18(1):38. 10.1186/s12939-019-0934-1 30819193 PMC6394065

[22] Whitlock EP , Orleans CT , Pender N , Allan J . Evaluating primary care behavioral counseling interventions: an evidence-based approach. Am J Prev Med 2002;22(4):267–84. 10.1016/S0749-3797(02)00415-4 11988383

[23] Neupane D , McLachlan CS , Mishra SR , Olsen MH , Perry HB , Karki A , Effectiveness of a lifestyle intervention led by female community health volunteers versus usual care in blood pressure reduction (COBIN): an open-label, cluster-randomised trial. Lancet Glob Health 2018;6(1):e66–73. 10.1016/S2214-109X(17)30411-4 29241617

[24] Jafar TH , Gandhi M , de Silva HA , Jehan I , Naheed A , Finkelstein EA , A community-based intervention for managing hypertension in rural South Asia. N Engl J Med 2020;382(8):717–26. 10.1056/NEJMoa1911965 32074419

